# Cloning and characterization of intermediate Homer1E of human skeletal muscle

**DOI:** 10.1007/s10974-026-09738-x

**Published:** 2026-08-27

**Authors:** Sandra Furlan, Marcello Carotti, Ahmed Samaha, Giovanni Minervini, Eylem Emek Akyurek, Emanuela Dazzo, Paola Lorenzon, Dieter Blottner, Michele Salanova, Dorianna Sandonà, Pompeo Volpe

**Affiliations:** 1 Istituto di Neuroscienze del Consiglio Nazionale delle Ricerche, Sezione di Padova, Padova, Italy; 2 Dipartimento di Scienze Biomediche, Università di Padova, Padova, 35123 Italy; 3 Dipartimento di Biomedicina Comparata e Alimentazione, Università di Padova, Legnaro (Padova), 35020 Italy; 4 Dipartimento di Scienze della Vita, Università di Trieste, Trieste, 34128 Italy; 5 Neuromuscular System, Institute for Integrative Neuroanatomy, Center of Space Medicine Berlin, Charité - University Medicine Berlin, Berlin, Germany; 6 Neuromuscular Signaling, Institute for Integrative Neuroanatomy, Center of Space Medicine Berlin (ZWMB), Charité Universitätsmedizin Berlin, 10115 Berlin, Germany

**Keywords:** HOMER, Cloning, Protein multimerization, Signal transduction

## Abstract

HOMER proteins are scaffolding proteins critically involved in intracellular signaling, calcium homeostasis, receptor trafficking and synaptic plasticity. Three human HOMER genes (*HOMER1*,* HOMER2*,* HOMER3*) are expressed in both neurons and skeletal muscle fibers. HOMER1 long isoforms multimerize via their carboxy-terminal coiled-coil domain, forming signaling clusters with postsynaptic density proteins. Despite growing evidence of HOMER1 relevance in skeletal muscle physiology, molecular data on human muscle remain scarce. This study investigates the expression of alternatively spliced HOMER1 transcripts in human skeletal muscle, with focus on a previously uncharacterized intermediate isoform, HOMER1E. Human skeletal muscle biopsies (Soleus and Vastus Lateralis) and cerebellum were analyzed by RT-PCR and droplet digital PCR (ddPCR) to quantify HOMER1 transcript variants. HOMER1E cDNA was cloned and expressed in HEK293 cells alongside full-length HOMER1. Protein stability was assessed using cycloheximide chase assays. Degradation pathways were investigated with MG-132 (proteasome inhibitor) and Bafilomycin A1 (autophagy inhibitor). Protein–protein interactions were evaluated by co-affinity purification and confocal immunofluorescence. Structural modeling employed AlphaFold-Multimer and DeepCoil predictions. Three HOMER1 transcripts (HOMER1, HOMER1H, HOMER1E) were detected in human skeletal muscle, with HOMER1E representing ~ 0.2% of total HOMER1 transcripts, as determined by ddPCR. HOMER1E encodes a 224 aa, 25.8 kDa protein which lacks exons 4–6 (including the autoinhibitory P-motif) but retains both N- and C-termini. In HEK293 cells, HOMER1E protein was highly unstable, primarily degraded via autophagy, while co-expression with HOMER1 significantly stabilized it. Co-affinity purification and immunofluorescence confirmed direct HOMER1–HOMER1E interaction. Structural modeling predicted a coiled-coil–mediated antiparallel heterodimer interface. HOMER1E is a minor, unstable HOMER1 isoform in human skeletal muscle with no murine counterpart. Its interaction with full-length HOMER1 via the conserved coiled-coil domain, combined with its rapid autophagic turnover, suggests a regulatory role in modulating multimeric HOMER1 scaffolding under specific physiological or developmental conditions.

**Supplementary Information:**

The online version contains supplementary material available at https://doi.org/10.1007/s10974-026-09738-x.

## Introduction

HOMER proteins constitute a family of scaffolding proteins that participate in intracellular signaling, particularly in neurons. They are essential for nervous system function since they regulate activity and localization of different receptors and channels, operating as scaffolding proteins at the post-synaptic density, thereby affecting calcium signaling, receptor turnover, synaptic plasticity, and synaptogenesis — particularly at glutamatergic synapses, where they also influence spine morphogenesis (Brakeman et al. 1997).

Three different HOMER genes have been identified in humans: *Homer1*,* Homer2, Homer3 *and, each of which yields numerous splice variants (Shiraishi-Yamaguchi and Furuichi 2007).

Beyond their synaptic roles, HOMER proteins have been implicated in a broad spectrum of neuropsychiatric conditions, including Autism Spectrum Disorders, schizophrenia, depression, anxiety, addiction and epilepsy (Szumlinski et al. 2006, Yoon et al. 2021), underscoring their importance as key regulators of signal transduction across multiple physiological and pathological contexts (Klugmann et al. 2005).

HOMER1, or long isoform, is known to be constitutively expressed in mammals (Shiraishi-Yamaguchi and Furuichi 2007) and consists of two major domains: the amino-terminal (1–175 amino acids) and the carboxy-terminal region (176–346 amino acids). The N-terminal region is highly conserved and has been named the conserved region of HOMER1 (CRH1). This region includes the Enabled/vasodilator-stimulated phosphoprotein (Ena/VASP) homology 1 (EVH1) domain and the proline-rich, autoinhibitory P-motif (138-Ser-Pro-Leu-Thr-Pro-142), present in both mouse and human HOMER1 proteins. The carboxy-terminal domain is a self-assembly, multimerization domain containing a coiled-coil (CC) structure and a leucine zipper motif. The three-dimensional organization of the HOMER CC-domain has been resolved by X-ray crystallography, revealing an antiparallel dimer that further assembles into tetrameric scaffolds through interactions between paired coiled-coils (Hayashi et al. 2009). HOMER1a, the first to be isolated (in the mouse), is known as the short isoform or immediate early gene (IEG) product because its expression is induced by synaptic activity in response to seizure or long-term potentiation (Brakeman et al. 1997). A carboxy-terminal splicing variant very similar to *HOMER1a*, Ania3, was also identified as an IEG induced by dopaminergic stimulation.

Multimers of HOMER1 proteins, assembled through their C-terminal CC-domains, are thought to form protein clusters with other postsynaptic density proteins, which are bound through the amino-terminal domains. Such HOMER1-mediated clusters probably regulate signal transduction and facilitate cross-talk between target proteins. The carboxy-terminal domain of HOMER1 isoforms mediates both homophilic and heterophilic interactions with different members of the family. The EVH1-like domain interacts with proline-rich sequences, i.e., Pro-Pro-x-x-Phe motifs that are found in many targets or ligand proteins, such as metabotropic glutamate receptor 1 (mGluR1), IP_3_ receptor type 1/3, RyR1 and TRPC1 (Bottai et al. 2002; Brakeman et al. 1997; Shiraishi et al. 2004; Tu et al. 1998; Yuan et al. 2003; Xiao et al. 1998). The CRH1 interacts with the neighboring CRH1s by intermolecular binding of the P-motif to the EVH1 domain at a site partly overlapping that used for Pro-Pro-x-x-Phe binding (Irie et al. 2002). Given that HOMER1 binding to mGluR1 induces its homo-multimerization (Brakeman et al. 1997), it is assumed that there are two types of binding of the HOMER1 EVH1 domain, i.e., binding to the Pro-Pro-x-x-Phe of the target protein and binding to the P-motif, favoring and blocking homo-multimerization, respectively (Shiraishi-Yamaguchi and Furuichi 2007).

Short HOMER isoforms lacking the carboxy-terminal domain are expressed in an activity-dependent manner as IEG products and act as dominant-negative regulators by disrupting HOMER1 clusters.

HOMER proteins were first identified and characterized in neurons but there is growing evidence that HOMER proteins play important roles in skeletal muscle as well (Salanova et al. 2002; Sandonà et al. 2000; Soloviev et al. 2000). It has been shown that HOMER proteins regulate the myogenic differentiation program (Bortoloso et al. 2006), the trophic state of the muscle (Salanova et al. 2011; Salanova et al. 2013) and the open probability (P_o_) of several ion channels (Feng et al. 2002). In particular, HOMER1 plays a specific role in skeletal muscle signal transduction, particularly in the regulation of Ca²⁺ release from the SR. HOMER proteins bind a proline-rich motif of the IP₃ receptor (Tu et al. 1998) that is also present in RyR1, the predominant Ca²⁺-release channel of skeletal muscle. Direct association of HOMER1c with RyR1 has been established by immunoprecipitation and GST pull-down, and although HOMER1 does not appear to be an integral part of the triadic complex (Volpe et al. 2004), it clearly associates with the latter. HOMER1c has been reported to influence RyR1 gating kinetics in vitro (Feng et al. 2002, Hwang et al. 2003) and to participate in the control of Ca²⁺ sparks (Ward et al. 2004) and Ca²⁺ release (Feng et al. 2002, Salanova et al. 2013); multimeric HOMER1c increases the spontaneous frequency of Ca²⁺ sparks in frog skeletal muscle fibers (Ward et al. 2004) and raises the P_o_ of RyR1 without changing single-channel conductance (Hwang et al. 2003). Several mechanisms have been proposed for this effect (Feng et al. 2002), including regulation of cooperativity among channel protomers, cross-linking of RyR1 oligomers to facilitate coupled gating (Marx et al. 1998), and cross-linking of RyR1 with the α1ₛ-DHPR to enhance EC-coupling efficiency in a manner already described for mGluR1 and IP_3_R (Tu et al. 1998, Xiao et al. 1998).

However, very few data are available on human skeletal muscle, particularly with respect to HOMER molecular physiology (Salanova et al. 2011). The present study shows the existence of HOMER1 isoforms in human skeletal muscle, encoded by alternatively spliced mRNA variants, quite different from those of murine counterparts. Moreover, we report a new alternatively spliced transcript of *HOMER*1 in human skeletal muscle: neither long nor short (H), but intermediate (E). As judged by ddPCR, *HOMER1E* is a very minor component of *HOMER1* transcripts whose translation product, at least in HEK293 cells, appears to be highly unstable because of active autophagy. Although HOMER1E function remains to be established in human skeletal muscle, its proven ability to interact with HOMER1, probably via the conserved CC domain, suggests a regulatory role in multimeric HOMER1 scaffolding.

## Methods

### Human and murine models

Human skeletal muscle biopsies from *Soleus* (SOL) and *Vastus lateralis* (VL) muscles were taken under local anesthesia from the left leg of each subject. Each biopsy was snap frozen in liquid nitrogen and stored at − 80 °C until use for molecular analysis. SOL biopsies were obtained from astronauts before the space flight (Blottner et al. 2024), VL biopsies from young adult males enrolled in a bed rest campaign at T0 (Lechado i Terradas et al. 2018). Human cerebellum tissue was obtained from autopsy on subjects with no recorded neurological alterations (Furlan et al. 2023).

Cerebellum (CBL), brain areas (CBR) and skeletal muscles, SOL and *Extensor Digitorum Longus* (EDL), were collected from 3-month-old male C57 mice. Such mice were provided by the in-house breeding facility at the Vallisneri complex of the University of Padova.

### RNA, PCR and sequencing

Total RNA was extracted from tissues with trizol^®^ method following the manufacturer’s instructions and including a glycogen co-precipitating step. Quantification and purity of RNA were assessed using the A260/A280 absorption method. Reverse transcription was performed on 1 µug of total RNA using the Superscript Vilo^®^ kit (Life Technologies-Thermo Fisher Scientific, Monza, Italy) or Reliance^®^ kit (Bio-Rad Laboratories, Hercules, CA, USA). NCBI, Uniprot, and Ensembl databases were used to search for human *HOMER1* transcripts. The NCBI database identifiers for the HOMER1 gene transcripts are as follows: HOMER1 Variant 1: NM_004272.5; HOMER1 Variant 2: NM_001277077.1; HOMER1 Variant 3: NM_001277078.1. Specific primers were designed using Primer3 software (http://frodo.wi.mit.edu/, Whitehead Institute for Biomedical Research) and their thermodynamic specificity was determined using BLAST sequence alignment (NCBI) and vector NTI^®^ software (ThermoFisher Scientific, Waltham, MA, USA).

The cDNA was used as template to amplify the Homer1 transcripts by PCR with the primers listed in Table 1 S. Briefly, cDNA was amplified in a 25 µl reaction mixture containing 1 U of Taq Polimerase with its buffer and 1,5 mM MgCl2 (Life Technologies-Thermo Scientific, Waltham, MA, USA), 100 µM of each dNTP, 0.4 µM of each primer. PCR amplification was performed for 45 cycles (94 °C for 30 s, annealing at proper temperature for 30 s, and 72 °C 30 s) on MyCycler (Bio-Rad, Hercules, CA, USA).

Aliquots of the PCR products obtained were analyzed by agarose gel electrophoresis using 1.8% agarose gels in 1 × TBE buffer. PCR products were excised and directly sequenced by BMR genomics (Padova, Italy). Sequences alignment was carried out using ClustalW2 (http://www.ebi.ac.uk/Tools/msa/clustalw2/).

For PCR on mouse cDNAs, all the primers used are listed in Table 2 S.

### ddPCR: absolute quantification of HOMER1 expression

For ddPCR, the reaction was prepared by mixing 2X QX200™ ddPCR™ EvaGreen Supermix (Bio-Rad Laboratories, Hercules, CA, USA), forward and reverse primers, RNase free-water and cDNA. The reaction mix was used to generate droplets with the QX200 droplet generator (Bio-Rad Laboratories, Hercules, CA, USA). After droplet generation, samples were transferred to a 96-well plate, sealed and amplified in a T100 Thermal Cycler (Bio-Rad Laboratories, Hercules, CA, USA) with following settings: 95◦C for 5 min, 45 cycles of 96◦C for 30 s and proper annealing temperature for 1 min, 4◦C for 5 min followed by 90◦C for 5 min. A ramp rate of 2◦C/s was used for all steps. Finally, the samples were analyzed in the QX200 Droplet Reader (Bio-Rad Laboratories, Hercules, CA, USA) and the results, including positive and negative droplets, were analyzed with QXMgrStandard™ (Bio-Rad Laboratories, Hercules, CA, U.S.A.).

Primers, listed in Table 1 S, were designed in-house and purchased from Invitrogen (Carlsbad, CA, USA).

### Plasmids, cell cultures, transfection and treatments

Isolation and purification of total RNA from adult SOL skeletal muscle were obtained using trizol^®^ method. Total RNA was converted into cDNA using a cDNA synthesis kit (ThermoFisher Scientific, Waltham, MA, USA). The primers used to amplify the cDNA corresponding to *HOMER1* (b) cDNA were as follows:

FW-Full length 5′-GGAAGGATCCATGGGGGAACAACCTATCTTCAG-3′ and

RV-Full length 5′-AGTTTCTAGATTAAGCGTAATCTGGAACATCGTATGGGTAGCTGCATTCTAGTAGCTTG GC-3′ for forward and reverse reactions, respectively.

BamHI and XBaI restriction sites were added to facilitate subsequent subcloning in the pCS2 + plasmid mammalian expression vector and, in separate constructs, either a 6×His-tag sequence was added at the 5′ end or an HA-tag sequence at the 3′ end. Two singly tagged HOMER1 constructs were thus generated - HOMER1-His and HOMER1-HA -, no construct carrying both tags. The cDNA corresponding to *HOMER1E* was amplified from the full length by the fusion of two fragments (omitting the exons 4–6); primers were as follows (5’−3’):

F-Homer 1E GGAAGGATCCATGCATCACCATCACCATCACGGGGAACAACCTATCTTCAGCA.

R-Inner GTTCAGTCACTTTCGAAAGATGATGCT.

F-Inner CTTTCGAAAGTGACTGAACTTGAATG.

R-Homer 1E AGTTTCTAGATTAGCTGCATTCTAGTAGCTTGGC.

The fused fragment was cloned into the pCS2 + plasmid mammalian expression vector using the same restriction sites and including, as for HOMER1 and again in separate constructs, either a 6×His-tag sequence at the 5′ end or an HA-tag sequence at the 3′ end, yielding two singly tagged HOMER1E constructs (HOMER1E-His and HOMER1E-HA). In all four constructs the 6×His tag was located at the 5′ (N-terminal) end and the HA tag at the 3′ (C-terminal) end; no construct carried both tags. All constructs were verified by sequencing.

For transient expression, HEK293 cells were seeded at 50,000 cells/cm^2^ density on poly-D-Lysine coated plates and transfected the day after seeding with TransIT293 (Mirus Bio, Madison, WI, USA), according to manufacturer’s instructions. After 36 h, cells were washed twice with ice cold PBS and lysed with RIPA buffer (Tris-HCl 50 mM pH 7.5, NaCl 150 mM, NP-40 1% v/v, sodium deoxycholate 0.5% w/v, SDS 0.1% w/v) supplemented with complete protease inhibitor (Sigma-Aldrich, St. Louis, MO, USA) for WB analysis.

To test protein stability, HEK293 cells were incubated with cycloheximide 100 µg/ml for 0, 0.2, 1, 2, 4 and 6 h. At each time point, cells were lysed with RIPA buffer supplemented with complete protease inhibitor and lysates were stored at −80 °C for future WB analysis.

To verify which protein degradation pathway was involved, transfected HEK293 cells were treated for either 8 h with 10 µM MG132 (Selleckchem S2619, Houston, TX, USA) or 24 h with either 50 or 100 nM Bafilomycin A1 (Selleckchem S1413, Houston, TX, USA): ensuing lysates were analyzed by WB.

To test interaction between Homer1 and HOMER1E, HEK293 cells were transfected with both constructs; after 48 h, cells were lysed with CHAPS buffer (Tris Hcl pH 7.5 50 mM, NaCl 150 mM, CHAPS 0.5%, imidazole 10 mM) supplemented with complete protease inhibitor. Equal amounts of total protein lysates were incubated overnight with 30 µl of a Ni-NTA agarose resin (Qiagen, Hilden, Germany), washed three times with CHAPS buffer containing 20 mM imidazole, and eluted with Laemmli’s sample buffer containing 250 mM imidazole. Both input and recovered proteins were analyzed by WB.

### Real time comparative PCR

Total RNA was extracted from HEK293T cells, transfected with the same amount of either HOMER1 or HOMER1E constructs, by using TriSure reagent (Meridian Bioscience, Cincinnati, OH, USA) according to the manufacturer’s instructions. 500 ng of total RNAs were retrotranscribed with the FastiSens cDNA synthesis kit (Meridian Bioscience, Cincinnati, OH, USA) according to manufacturer’s instructions. The relative quantification of full-length *HOMER1* and intermediate *HOMER1E* was done by real time qPCR using the HOMER1 FWD (pan HOMER1) 5’-TCCAAATTGACCCAAACACA-3’ and HOMER1 REV (pan HOMER) 5’- TTGCCTTTGAGCCATCTAAA − 3’.

β−2-microglobulin (β2M) was amplified as the reference gene using the following primers: B2M FWD primer 5’ -TATCCAGCGTACTCCAAGA-3’ and B2M REV primer, 5’-GACAAGTCTGAATGCTCCAC-3’. qPCR was performed in triplicate in IQ5 Thermal Cycler using Biorad iQ Sybr green supermix. The PCR parameters were: initial denaturation at 95 °C for 2 min followed by 40 cycles of 10 s at 95 °C and 30 s at 50 °C for acquisition of fluorescence signal.

A melting curve was made at the end of the final cycle for each sample to confirm the specificity of the amplified products. The efficiency of each amplification was determined and used for calculations. The relative abundance of HOMER1 and HOMER1E was determined with the delta-delta Ct method.

Data are expressed as means +/- SD of three experiments. Comparisons were made using the t test, with values of *P* < 0.05 considered statistically significant.

### WB Analysis

Protein concentration of the cell lysates prepared after HEK293 treatments was determined by the BCA assay (Thermo Fisher Scientific, Waltham, MA, USA) according to the manufacturer’s instructions.

Proteins were then resolved by SDS-PAGE, blotted onto a nitrocellulose membrane and probed with selected antibodies: mouse monoclonal anti His tag (1B7G5, Proteintech, Rosemont, IL USA) rabbit polyclonal anti HA (ab9110, Abcam, Cambridge, UK), rabbit polyclonal anti Homer 1 (AV40182, Sigma-Aldrich, St. Louis, MO, USA), mouse monoclonal anti-tubulin (66240, Proteintech, Rosemont, IL USA) HRP-conjugated secondary anti-rabbit and anti-mouse antibodies were from Sigma Aldrich (St-Louis, MO, USA), as described by Furlan et al. (Furlan et al. 2023). The antibodies used to detect HOMER1 and HOMER1E by Western blotting in each figure were as follows: anti-His (HOMER1) and anti-HA (HOMER1E) in Fig. 4; anti-HA (both isoforms) in Figs. 6, 7 and 9; anti-Homer1 (full-length HOMER1) and anti-HA (HOMER1E) in the co-affinity purification of Fig. 8; and, for immunofluorescence, anti-His (HOMER1) and anti-HA (HOMER1E) in Fig. 10.

## Immunofluorescence analysis

HEK293 cells were grown on 13 mm glass coverslips. After transfection and treatment, cells were gently washed with PBS, pH 7.4, and fixed with 4% paraformaldehyde for 15 min at room temperature. Cells were washed with PBS and then permeabilized in 0.5% Triton X-100 in PBS. Anti-HA1 and anti-HIS tag primary antibodies were used in 1% BSA/PBS solution. After the incubation period, cells were gently washed with PBS and incubated with 2 µg/mL of either Alexa Fluor 568 red or Alexa Fluor 488 green secondary antibodies (ThermoFisher Scientific, Waltham, MA, USA). Nuclear morphology was characterized by staining with 1 µg/mL Hoechst 33342 (ThermoFisher Scientific, Waltham, MA, USA). Glass coverslips were closed with mowiol (Sigma-Aldrich, St. Louis, MO, USA) and observed in Leica TCS SP5 II confocal microscope using a WLL white laser (Leica) equipped with a PlanApo 100 × (numerical aperture 1.4) oil immersion objective equipped with Leica HyD hybrid detector.

Immortalized human myoblasts (AB678) were obtained from the Human Cell Culture Platform of the Myology Institute in Paris (Zhu et al. 2007). AB678 myogenic cells were cultured in Ham’s F-12 Nutrient Mix medium supplemented with 20% fetal bovine serum (Thermo Fisher Scientific, Waltham, MA, USA), insulin (10 µg/mL), fibroblast growth factor (5 ng/mL), and epidermal growth factor (5 ng/mL) (Sigma-Aldrich, St. Louis, MO, USA). For immunofluorescence, myoblasts were seeded onto 13 mm glass coverslips and transfected the following day using Lipofectamine 3000 (Thermo Fisher Scientific, Waltham, MA, USA) according to the manufacturer’s instructions. After 48 h, myoblasts were processed for immunofluorescence as described above for HEK293T cells.

### In silico methods

**Fig. 1:**
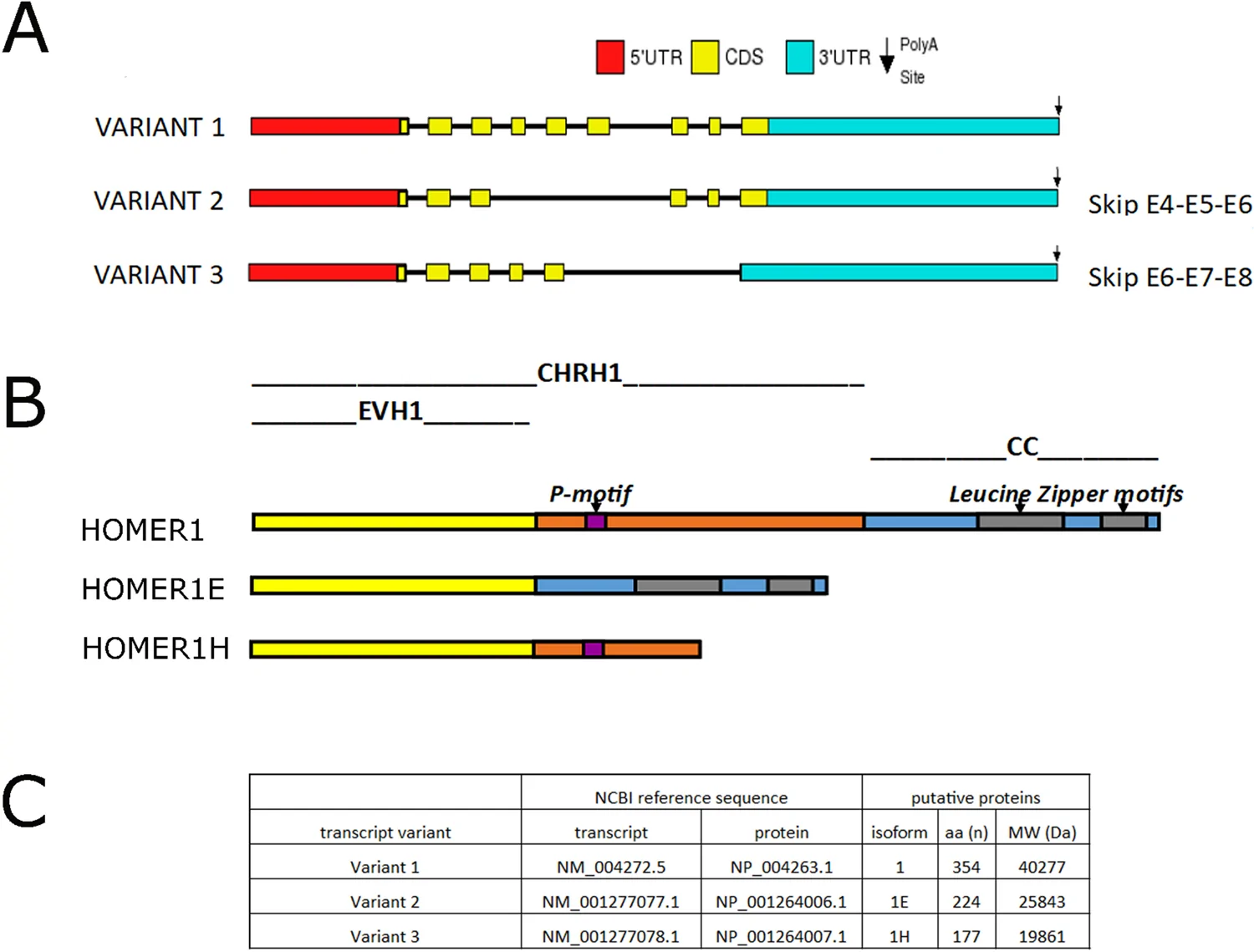
Schematic representation of mRNAs and proteins from human *HOMER1* gene. (**A**) The *HOMER1* gene is located in chromosome 5 and contains 9 exons. Alternative splicing results in 3 mRNA transcripts (variants 1, 2 and 3) as reported in the NCBI RefSeq database (https://www.ncbi.nlm.nih.gov/ gene). (**B**) Primary structures of HOMER1 protein isoforms. The position of functional features is shown on the top. CHRH1: conserved region of HOMER1; EVH1: Enabled/vasodilator-stimulated phosphoprotein (Ena/VASP) homology 1; CC: coiled-coil structure; P-motif (SPLTP), proline-rich motif, specific to the mammalian Homer1 subfamily. In grey, leucine-zipper motif. Numbers on the right represent protein length in amino acid (kDa) (modified from Shiraishi-Yamaguchi, Y. and Furuichi, T. (2007) Ref. 1). (**C**) List of *HOMER1* transcripts reported in NCBI Reference Sequences database. The NCBI Reference Sequence number and the characteristics of the putative proteins are reported for each transcript and protein isoform

Amino acid sequences of Homer1 (UniProt: Q86YM7) and Homer1E (UniProt: Q86YM7-2) were obtained from the UniProt database (https://www.uniprot.org/). The monomeric structure of Homer1E was predicted using AlphaFold (Sandonà et al. 2000; Abramson et al, 2024) with default parameters. Structural confidence was evaluated through the pLDDT and PAE. Representative confidence maps are provided in Fig. 1S. The propensity of C-terminal helical region of Homer1E to form coiled-coil motif was evaluated using DeepCoil (neural-network-based coiled-coil predictor in Ref. 8) to identify coiled-coil forming residues and potential heptad registers. Predictions were compared with the corresponding region in canonical Homer1. Potential heterodimerization between Homer1E and canonical Homer1 was evaluated using AlphaFold-Multimer (Abramson et al. 2024), with models ranked by ipTM/pTM scores, interface quality, and structural plausibility of the coiled-coil arrangement using tool included with the software. Only the highest-confidence models were retained for analysis. All structural models were inspected using UCSF Chimera (Pettersen et al. 2004), which was also used to analyze the coiled-coil interface, visualize hydrophobic packing and electrostatic complementarity, and generate Fig. 11.

### Statistical analysis

Data are expressed as mean ± SEM. Statistical analysis on ddPCR was performed using Origin 8. Normality of data distribution was assessed with the Shapiro-Wilk test. Either unpaired t test or one way ANOVA followed by the Tukey post-hoc test was used for comparison of normally distributed data. Data are expressed as mean ± standard error of the mean (SE).

## Results

The NCBI database was analyzed in search of alternatively spliced transcripts of the human *HOMER1* gene. NCBI Reference Sequences (*RefSeq*) database for the human *HOMER1* gene (https://www.ncbi.nlm.nih.gov/gene/9456, based on chromosome 5, updated on November 25, 2025) reports 5 transcripts: 3 are validated transcripts named Variant 1, Variant 2 and Variant 3, whereas 2 are listed as Xm predicted model, X1 and X2; validated variants code for 3 validated protein isoforms, reported in uniprot (https://www.uniprot.org/uniprotkb/Q86YM7) and named HOMER1, HOMER1E and HOMER1H, respectively (Fig. 1C).

The first of such NCBI *RefSeq* transcripts corresponds to the long canonical *HOMER1* transcript variant1, which is composed of 9 exons and encodes a 354 aa long protein having a MW of about 40.3 kDa (Fig. 1). The other predicted variants correspond to alternatively spliced products.

Human Transcript Variant 3, also referred to as *HOMER1H*, lacks three consecutive coding exons (6–8) in frameshift, as compared to Variant 1. Thus, the ensuing protein isoform, as it happens for mouse HOMER1a, is endowed with a shorter and distinct C-terminus, as compared to the Variant 1 (HOMER1), with a MW of about 19,9 kDa (Fig. 1A–C).

Transcript Variant2, also called *HOMER1E*, lacks three consecutive coding exons (4–6) as compared to variant1. The missing segment is in-frame and the ensuing protein isoform has the same N- and C-termini but is shorter than isoform 1 (224 aa for a MW of about 25.8 kDa; Fig. 1A–C). In this variant, the P-motif is missing. No HOMER1E analog has been described in mouse and rat.

Despite the existence of genomic and proteomic databases concerning HOMER1, all literature data focus on the two long HOMER1 isoforms with the self-assembly CC domain and the two short HOMER1 isoforms without the CC domain. No data exist on the intermediate HOMER1E isoform. This paper investigates the expression of different spliced HOMER1 isoforms in human skeletal muscles using PCR and droplet digital PCR (ddPCR) with primers designed specifically for this study, as reported in Table 1 S.

### Analysis of mRNA

As shown in Table 1 S, primer set 1, 2 and 3 were specific for human Variant1 (*HOMER1* full length), Variant 2 (or *HOMER1E)* and Variant 3 (or *HOMER1H*) transcripts, respectively. The predicted PCR products ranged in size from 215 bp for Variant 1, to 305 bp for Variant 2, to 271 bp for Variant 3.

**Fig. 2:**
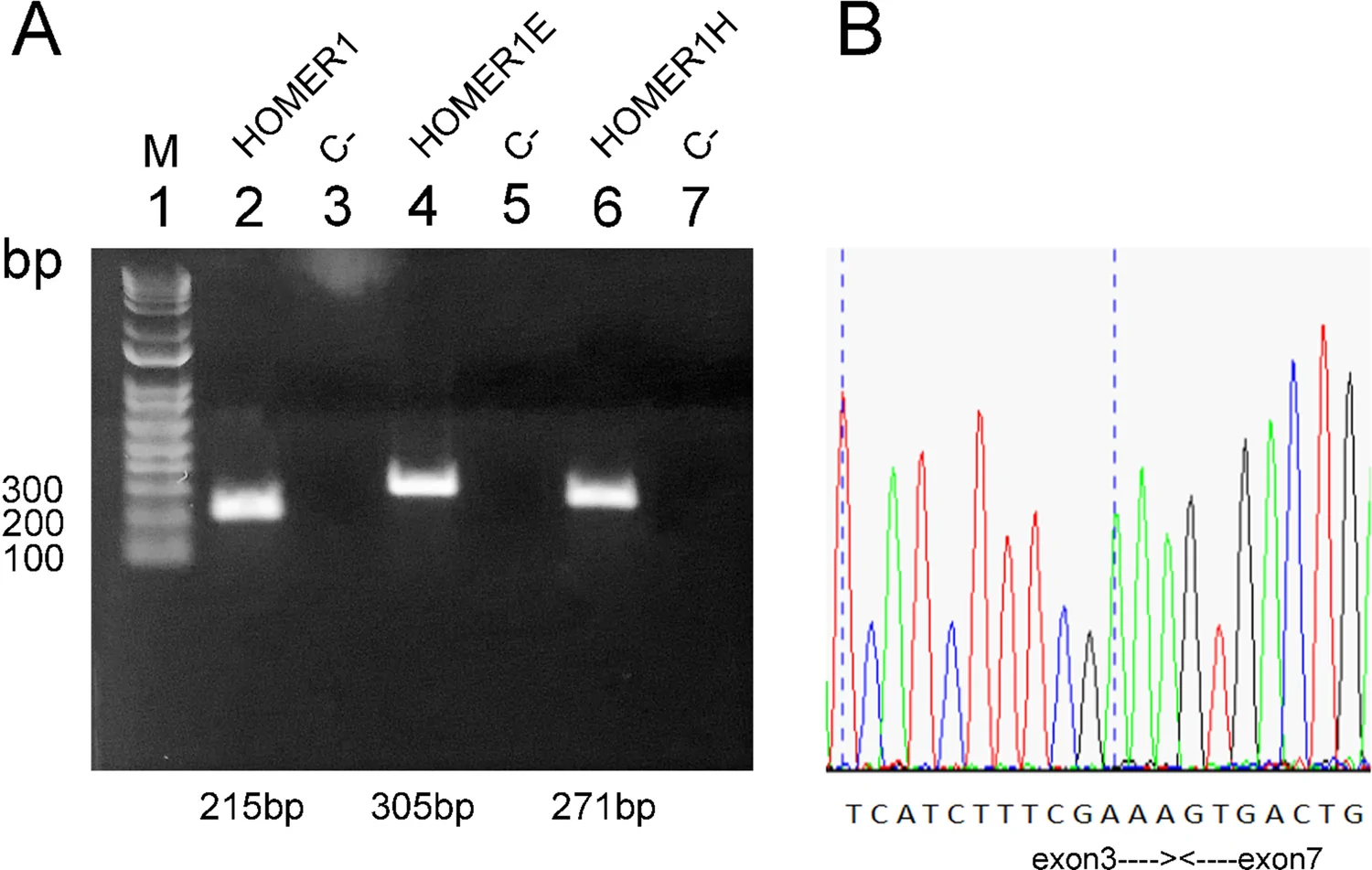
HOMER1 mRNA variants obtained by RT-PCR from Human SOL skeletal muscle using primer pairs listed in Table S1. (**A**) Agarose gel electrophoresis of RT-PCR HOMER1 amplified variants. All products obtained were sequenced to confirm their identity and the specificity of the reaction. Sizes of the expected bands are reported on the bottom. (**B**) Sequence analysis of the RT-PCR product for HOMER1E showing the alternative splicing event that results in the juxtaposition of Exon 3 and Exon 7

In a preliminary 35 cycles long PCR experiment, only Variant 1 transcript was detected in human SOL, a well characterized slow-twitch skeletal muscle (data not shown). In subsequent 45 cycles long PCR, on the same muscle, it was also observed the expression of both *HOMER1E and HOMER1H* alternative transcripts (Fig. 2), as reported in NCBI *RefSeq* database.

Since *HOMER1* has been detected in brain, total mRNA isolated from an autoptic human cerebellum (CBL) was used as positive control to validate the proper length of amplicons for *HOMER1*. The identical size of all the PCR products obtained from human CBL and skeletal muscles, as determined by agarose gel electrophoresis (Fig. 2S, panel A), confirmed the specificity of the primer pairs for each variant and the expression of the same variants in CBL and skeletal muscle samples.

The presence of only one transcript was confirmed for the long isoform of HOMER1 (FL or Variant1) in human muscles (Fig. 2, lane 2, and Fig. 2S, panel A, lanes 2 and 3). As expected, no 36 bp HOMER1c-analogous sequence encoded by rodent exon 6 nor DNA insertions at the expected position of human ESTs were found (Tadokoro et al. 1999).

The 305 bp fragment obtained from Variant 2 amplification (Fig. 2, lane 4) - with a forward primer targeting exon 2 and reverse primer spanning exon 3-exon7 junction of Variant 2 - confirmed that the *HOMER1E* transcript is expressed both in skeletal muscle and CBL (Fig. 2S, lanes 5 and 6). In addition, for Variant 2 a 480 bp long fragment, spanning from exon 3-exon7 junction to exon9, was also amplified to confirm the identity of Variant 2 (data not shown). No other band corresponding to non-specific amplification of other variants was detected.

In parallel, the corresponding mouse *HOMER1* transcripts were amplified by using specific primers listed in Table 2S. As shown in Fig. 2S, panel B, mouse *HOMER1C* was prevalent in brain areas (lanes 1, 2) whereas both *HOMER1B* and *1 C* were clearly detectable in muscles (lanes 3, 4). Both *HOMER1M* (lanes 5–8) and the short isoform *HOMER1S* (lanes 9–12) bands were clearly detected in both brain areas and skeletal muscles.

### Expression of HOMER1 transcripts

The relative expression of alternative HOMER1 transcripts was investigated by quantitative PCR. Since the expression of the alternatively spliced isoforms is very low, we failed to obtain reliable quantitation by qPCR, particularly in the case of *HOMER1E* (data not shown). Thus, a more powerful droplet digital PCR (ddPCR) approach was implemented in order to assess the contribution of each variant to the total pool of *HOMER1* transcripts in human skeletal muscles via absolute quantification of DNA copy numbers (Taylor et al. 2017).

**Fig. 3:**
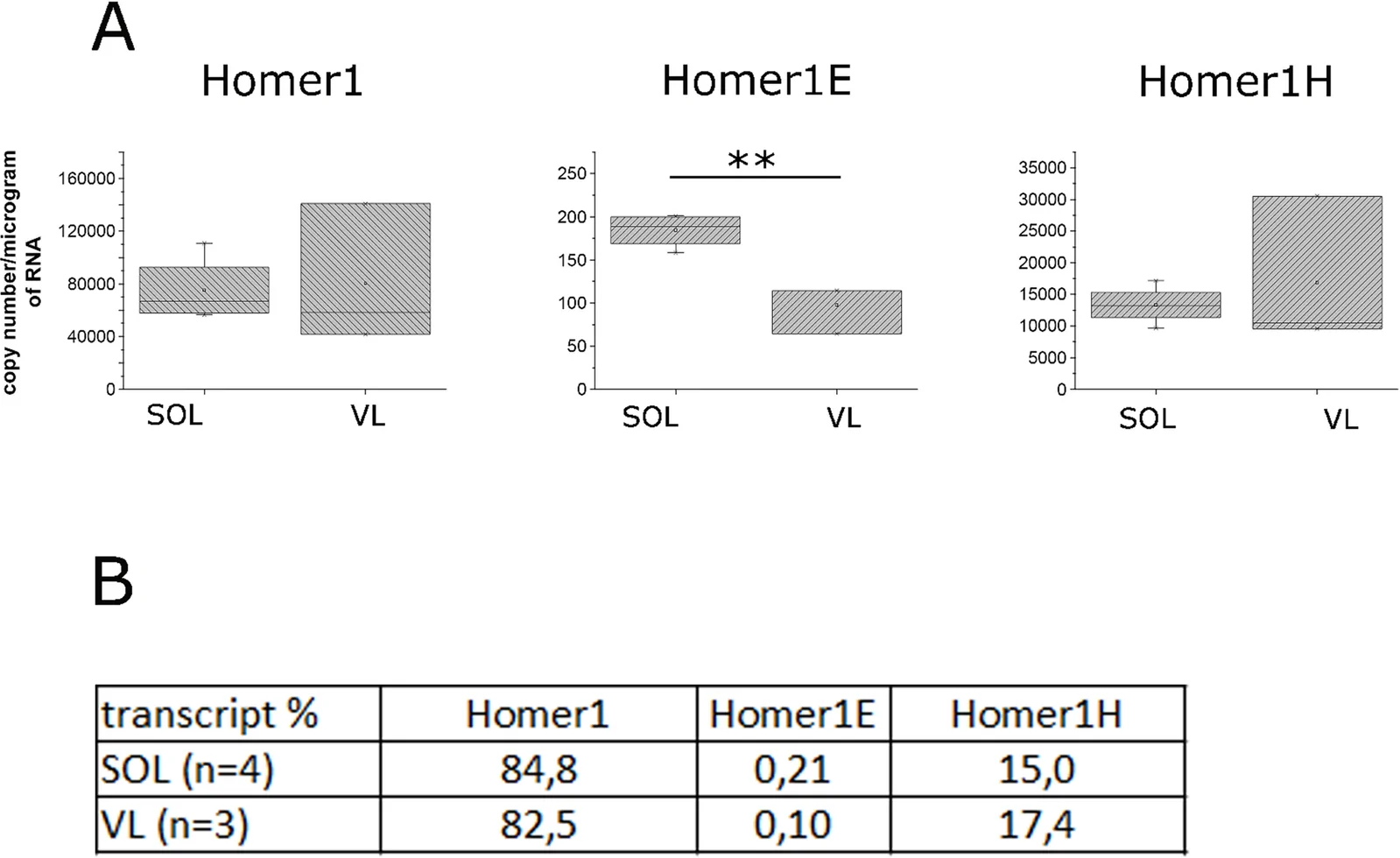
Absolute quantification of *Homer1* mRNA expression by ddPCR in human skeletal muscles. (**A**) *HOMER1*,* HOMER1E* and *HOMER1H* mRNA abundance was measured in total RNA extracts obtained from Soleus, *n* = 4, and VL, *n* = 3; transcript copy number/µg of total RNA, analyzed with comparisons test, are expressed as mean ± SEM. *p < 0.05; **p < 0.01, ***p < 0.001. (**B**) Percentage values referable to each alternatively spliced transcript

To this effect, analysis was carried out on SOL muscle, which is composed of mostly slow-twitch fibers, and *Vastus Lateralis* (VL), a mixed muscle composed of both fast- and slow-twitch fibers. As shown in Fig. 3A, the three transcripts were quantified by ddPCR, SOL data being less scattered. Data are also reported in Fig. 3B, the relative percentage being approximately of > 80, <20 and ~ 0.2 for *HOMER1*,* HOMER1H* and *HOMER1E*, respectively.

#### cDNA cloning of Human *HOMER1E*

The full-length cDNA of HOMER1 was obtained by RT-PCR from human skeletal muscle RNA. The intermediate *HOMER1E* cDNA was subcloned from the full-length *HOMER1* cDNA, by domain deletion PCR (see Methods for details), and its complete sequence is shown in Fig. 3S. For subsequent expression experiments (see below), each cDNA was expressed as two separate singly tagged constructs — one bearing a 6×His tag at the 5′ (N-terminal) end and one bearing an HA tag at the 3′ (C-terminal) end (four constructs in total; no construct carried both tags) — inserted into pCS2 + plasmids (see Methods for details).

### HOMER1E protein expression in HEK293 cells and biochemical characterization

Due to the very low abundance of the *HOMER1E* transcript in human tissues and in order to study the translated product of the human *HOMER1E*, overexpression experiments were carried out in mammalian HEK293 cells. pCS2 + plasmids were transiently transfected into HEK293 cells and resulting cell lysates were then analyzed by WB, using specific antibodies for the tags fused to either HOMER1 or HOMER1E proteins.

**Fig. 4:**
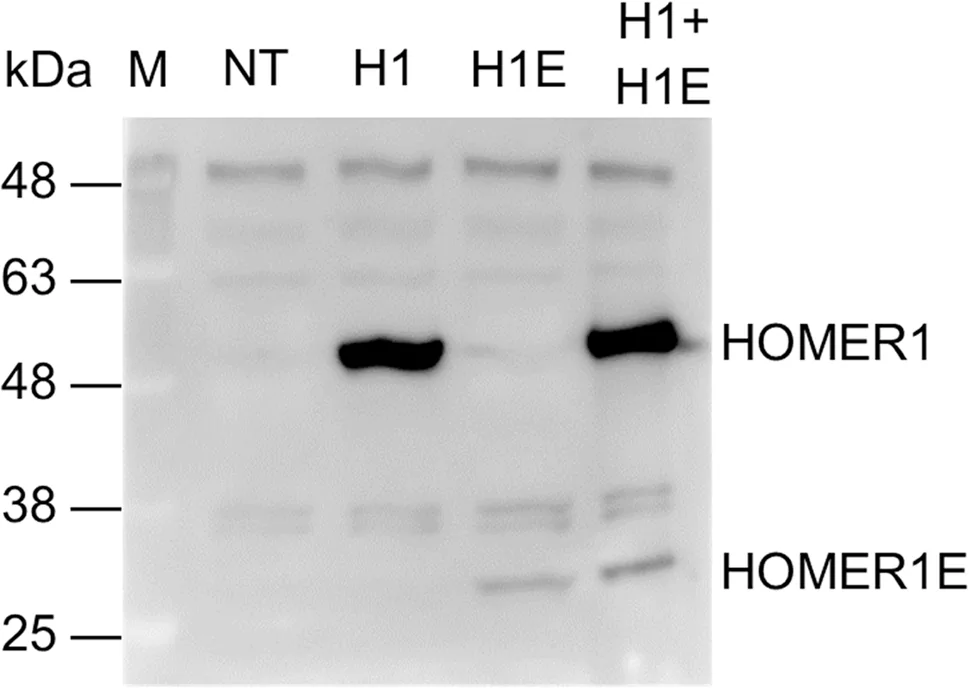
Expression of HOMER1 and HOMER1E in transfected HEK293 cells. pCS2 + plasmids were transiently transfected into HEK293 cells and resulting cell lysates were then analyzed by WB, using specific antibodies for the tags fused to either HOMER1 or HOMER1E proteins. Following WB, HOMER1 was detected as 40.2 kDa band, whereas HOMER1E was detected as a 25.8 kDa band. M=molecular weight markers, NT = non transfected

WB analysis revealed expression of both proteins, albeit at different levels. The HOMER1 isoform appeared as an intense 40.2-kDa band, whereas HOMER1E appeared as a faint 25.8-kDa band (Fig. 4). In co-transfection experiments (H1 + H1E), the amount of HOMER1E appeared to increase (Fig. 4).

**Fig. 5:**
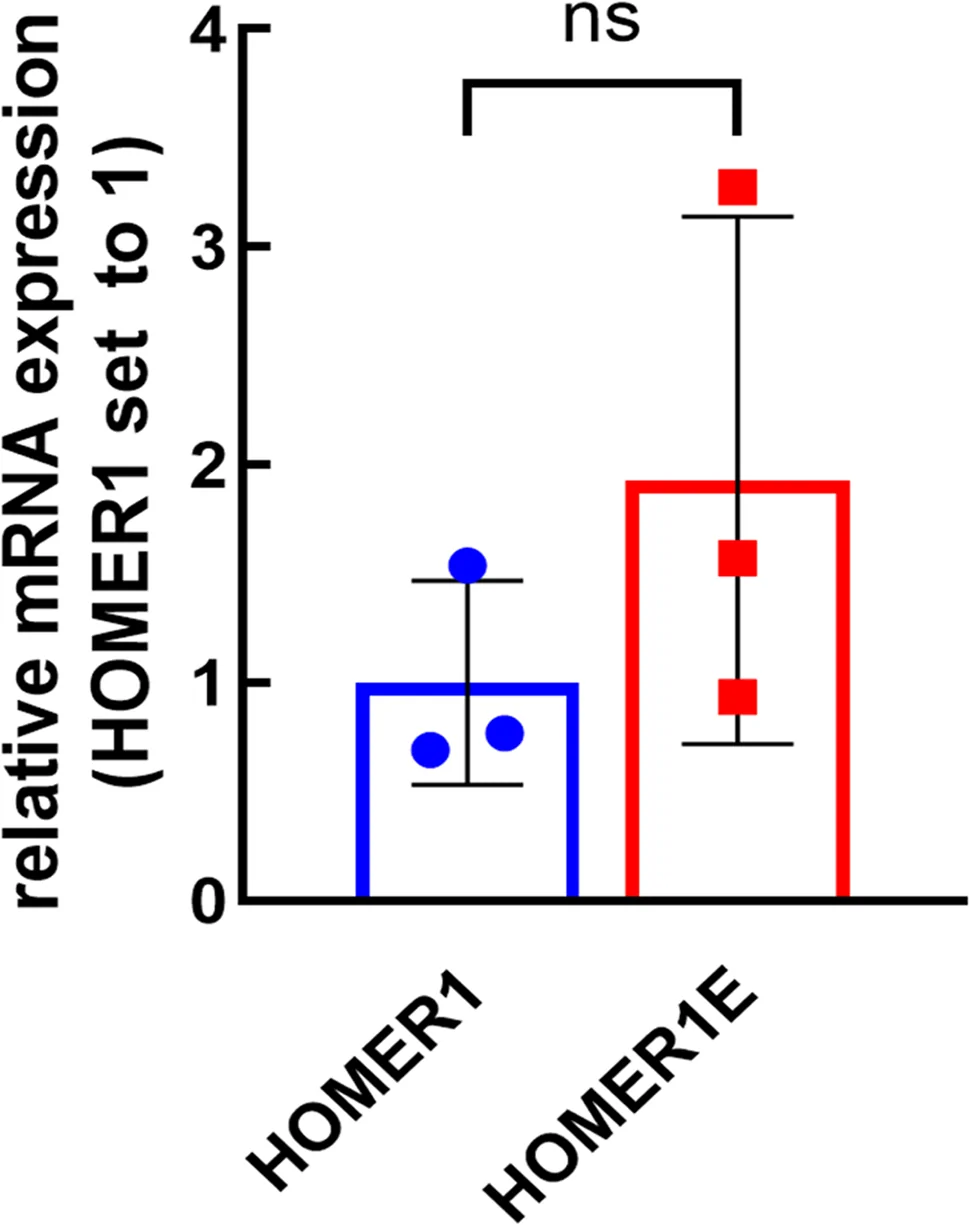
Relative expression of HOMER1 and HOMER1E mRNA by RT-PCR of transfected HEK293 cells. mRNA levels of both *HOMER1* and *HOMER1E* were measured by RT-PCR, are given as relative values and are expressed as mean ± SEM; ns, not significant, as judged by Student t-test

Because the levels of mRNA for HOMER1 and HOMER1E were similar after transfection (Fig. 5), it is reasonable to conclude that the HOMER1E protein is considerably less stable than the HOMER1 isoform.

**Fig. 6:**
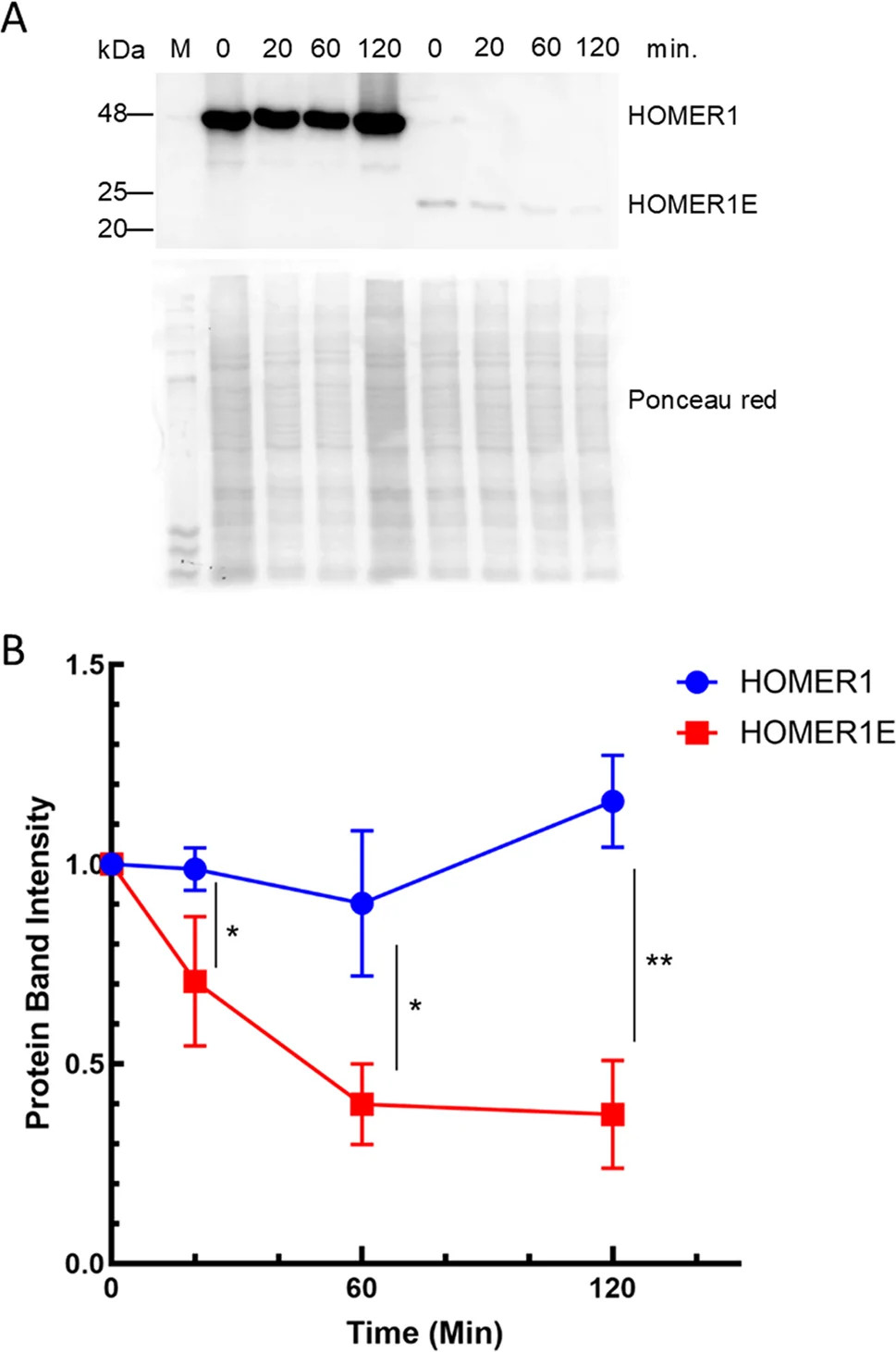
Effects of cycloheximide on HOMER1E stability in transfected HEK293 cells. Cell lysates were analyzed by SDS-PAGE and WB: upper panel, representative time course of HOMER1 and HOMER1E expression up to 120 min following 1 h treatment with cycloheximide. Lower panel: 4 replicates of experiments reported in the Upper panel were carried out and intensities of immunolabelled proteins referable to either HOMER1 or HOMER1E are given in arbitrary densitometric units and are expressed as mean ± SEM for *n* = 4; *p < 0.05; **p < 0.01, as judged by Student t-test

To estimate the half-life of HOMER1 proteins, a subsequent set of experiments was carried out in which HEK293 cells were transfected and treated with cycloheximide, a well-known inhibitor of protein translation, for one hour. This allowed the level of HOMER1 isoforms to be evaluated by WB (Fig. 6A). As shown in Fig. 6B, the amount of HOMER1 protein remained relatively constant over time, whereas HOMER1E levels decreased significantly, suggesting that HOMER1E is an unstable protein rapidly degraded by the cell.

**Fig. 7:**
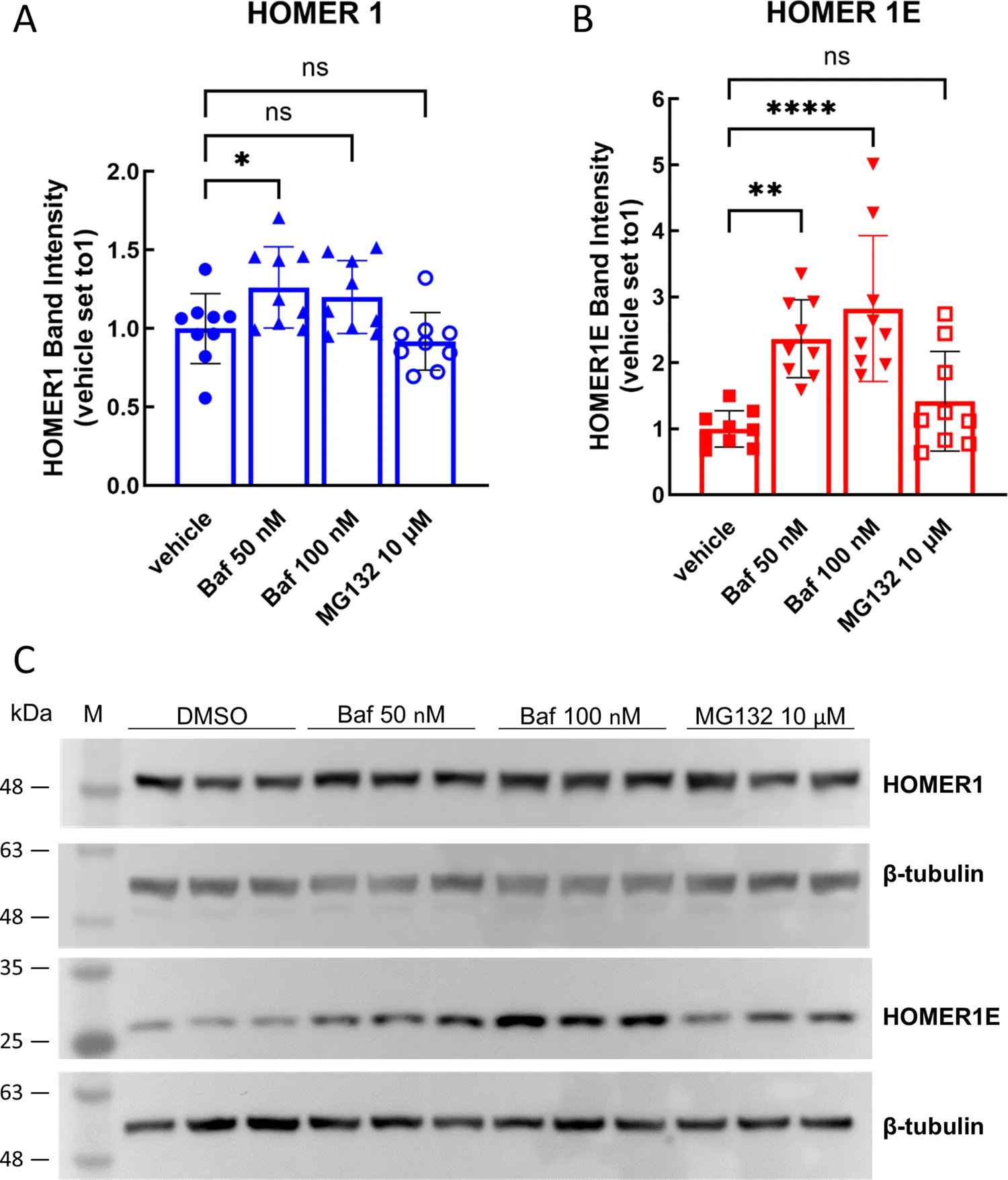
Effect of either MG-132 or bafilomycin A1 on HOMER1 isoforms stability in transfected HEK293 cells. (**A**) Homer1. Relative amounts of HOMER1 upon treatment with Bafilomycin A1 (Baf) or MG-132 were measured by densitometry, given as percentage vs. control (vehicle) and expressed as mean ± SEM for *n* = 9; *p < 0.05; ns, not significant, as judged by One way ANOVA followed by Tukey’s post hoc test. (**B**) Homer1E. Relative amounts of HOMER1E upon treatment with Bafilomycin A1 (Baf) or MG-132 were measured by densitometry, given as percentage vs. control (vehicle) and expressed as mean ± SEM for *n* = 9; **p < 0.01, ****p < 0.0001; ns, not significant, as judged by One way ANOVA followed by Tukey’s post hoc test. (**C**) Homer1. Representative experiment of WB analysis of HOMER1 expression upon treatment with either DMSO (vehicle), Baf or MG-132. Densitometric data referable to HOMER1 were normalized against those referable to β-tubulin. No lower-molecular-weight immunoreactive species attributable to partial proteolytic degradation of either HOMER1 or HOMER1E were detected on WB under any of the treatment conditions tested, consistent with autophagic-lysosomal degradation proceeding via bulk proteolysis rather than discrete, stable cleavage intermediates

To determine which catabolic pathway is responsible for HOMER1E degradation, HEK293 cells were transfected and treated with either MG-132, which blocks proteasomal degradation, or bafilomycin A1, which blocks lysosomal degradation and is also a proton pump inhibitor (Yamamoto et al. 1998). As shown in Fig. 7A and B, reporting the densitometric analysis of different WBs, MG-132 did not substantially affect the levels of both HOMER1 isoforms. On the other hand, bafilomycin greatly increased the amount of HOMER1E and only slightly affected the amount of HOMER1. This indicates that HOMER1E is primarily degraded through autophagy.

### HOMER1E interacts with HOMER1 and is protected from degradation

**Fig. 8:**
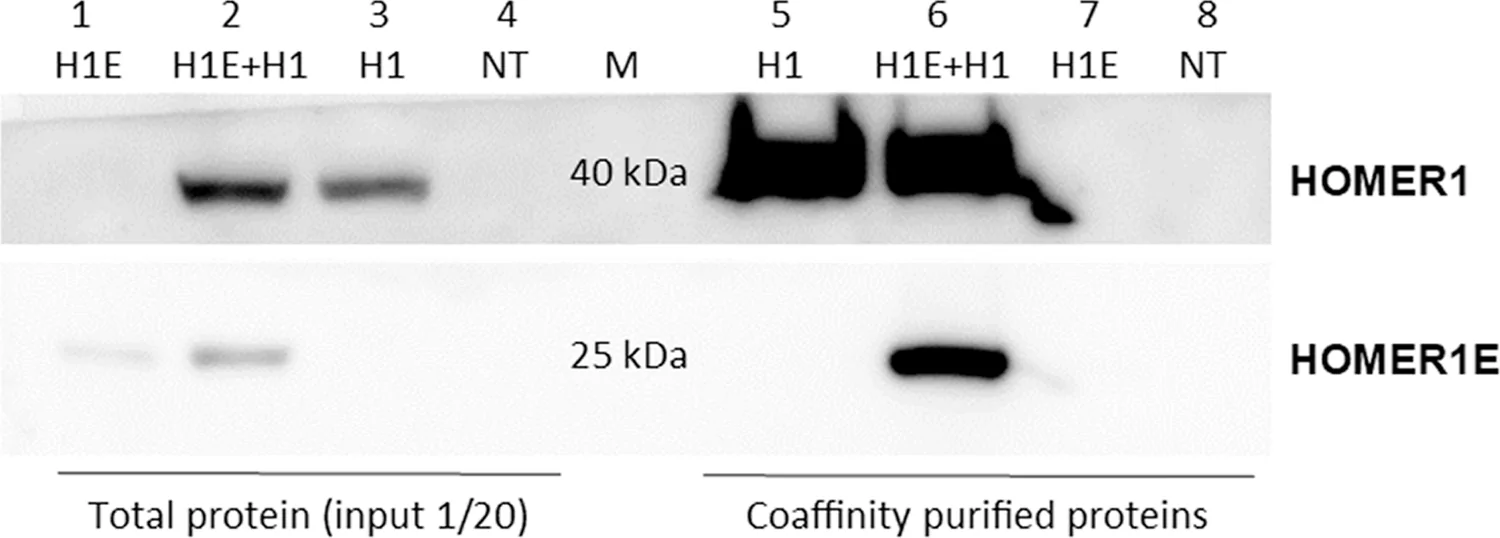
Co-affinity purification of Homer1 and Homer1E. HEK293 cells were either transfected with cDNAs encoding HOMER1E or co-transfected with equimolar amounts of cDNAs encoding either HOMER1 or HOMER1E. To transfect the same total amount of DNA and maintain a consistent DNA/transfection reagent ratio across all samples, an empty vector plasmid was added to the single transfection conditions. Lanes 1–4, input, 1/20 of total protein lysates obtained from either single transfected (H1, H1E) or co-transfected (H1 + H1E) cells. Following cell lysis, HOMER1 was affinity purified using the HIS-tag; lanes 5–8, co-affinity purified proteins from either single transfected (H1, H1E) or co-transfected (H1 + H1E) cells were identified by WB using anti-Homer1 antibody (for full-length HOMER1) and anti-HA antibody (for HOMER1E). NT, not transfected

The fundamental scaffolding function of HOMER proteins depends, at least in part, on specific homophilic interactions among different isoforms. To investigate this in HOMER1 and HOMER1E, HEK293 cells were either transfected with cDNAs encoding the long or intermediate isoform, or co-transfected with equimolar amounts of cDNA coding for both isoforms. Figure 8 (lanes 1–4 show the protein inputs of the experiment) demonstrates that lysates obtained from co-transfected HEK293 cells produced a higher concentration of HOMER1E compared to singly transfected cells (see lane 2 compared to lane 1). Following cell lysis, HOMER1 was isolated using the HIS-tag (Fig. 8, lanes 5–6). Alongside the full-length isoform, HOMER1E was recovered from the co-transfected cells. This indicates that, under the prevailing experimental conditions, HOMER1 and HOMER1E form a stable complex. The absence of a HOMER1E signal in lane 7 indicates that this isoform does not interact with the resin.

**Fig. 9:**
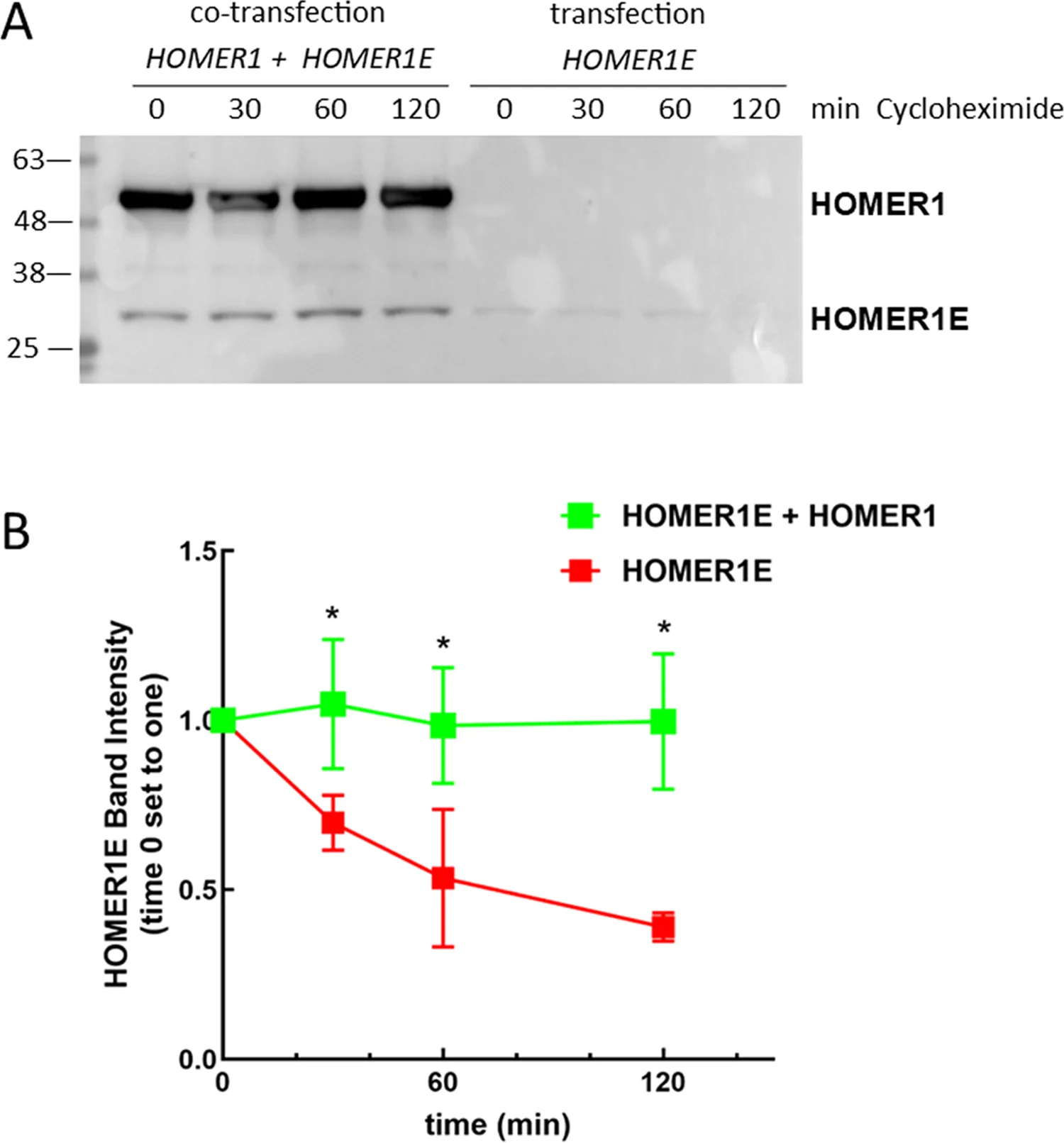
Stabilization of Homer1E following co-expression with Homer1. Experiments were carried out as described in legend to Fig. 6: HEK293 cells were either transfected with cDNAs encoding HOMER1E or co-transfected with equimolar amounts of cDNAs encoding either HOMER1 or HOMER1E. Time course of HOMER1 and HOMER1E expression was monitored up to 120 min following 1 h treatment with cycloheximide. (**A**) WBs of cell lysates probed with anti-HA antibody (detecting both HOMER1 and HOMER1E); (**B**) HOMER1E band intensity is given in arbitrary densitometric units and is expressed as mean ± SEM for *n* = 4. *p < 0.05, as judged by Student t-test

To further demonstrate the role of HOMER1 in stabilizing HOMER1E, HEK293 cells were treated with cycloheximide. The cells were transfected either with HOMER1E alone or with both HOMER1E and HOMER1. Figure 9 shows the time course of expression of both isoforms. Panel A depicts a representative WB, and Panel B shows the densitometric analysis from multiple experiments. The amount of HOMER1E degraded by about 50% within one hour when expressed alone. However, it was protected from degradation when co-expressed with HOMER1, likely due to an interaction between the two proteins.

**Fig. 10:**
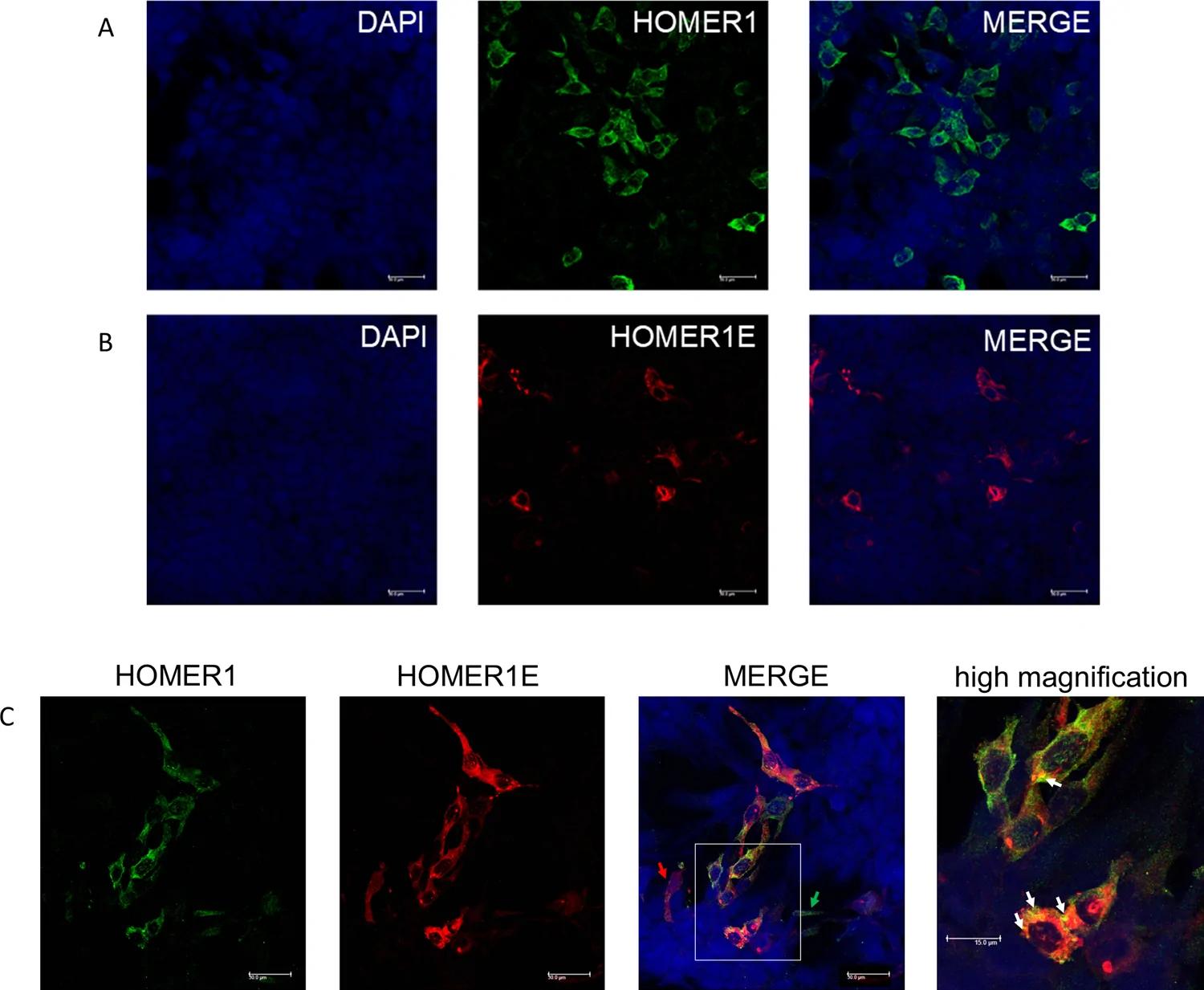
Confocal Immunofluorescence of transfected HEK293 cells. (**A**) HEK293 cells transfected with cDNAs encoding HOMER1. Scale bars, 30 μm. (**B**) HEK293 cells transfected with cDNAs encoding HOMER1E. Scale bars, 30 μm. (**C**) HEK293 cells co-transfected with equimolar amounts of cDNAs encoding either HOMER1 or HOMER1E. The red arrow in the MERGE panel indicates cells expressing only HOMER1E, whereas the green arrow points to cells expressing only HOMER1. Scale bars, 30 μm. In the high magnification panel, corresponding to the inset of MERGE panel, white arrows point to areas of colocalization. Scale bar, 15 μm

To further assess the interaction between HOMER1 and HOMER1E, HEK293 cells were transfected with the corresponding cDNAs or co-transfected with an equimolar amount of both constructs. The cells were then immunolabeled using antibodies that target the protein-specific tags, either His or HA. As shown in Fig. 10, several cells expressed HOMER1 (panel A) and HOMER1E (panel B). In co-transfection experiments (Panel C), partial colocalization of the green HOMER1 and red HOMER1E fluorescent signals was observed. This colocalization is more clearly seen at higher magnification (white arrows in the right panel of Fig. 10C).

Finally, in anticipation of a more comprehensive study in skeletal muscle fibers, we obtained preliminary evidence of exogenous expression of both HOMER1E and HOMER1, as well as their partial colocalization in human AB678 myogenic cells (Fig. 4S).

### Structural model of HOMER1E and coiled-coil driven dimerization with HOMER1

**Fig. 11:**
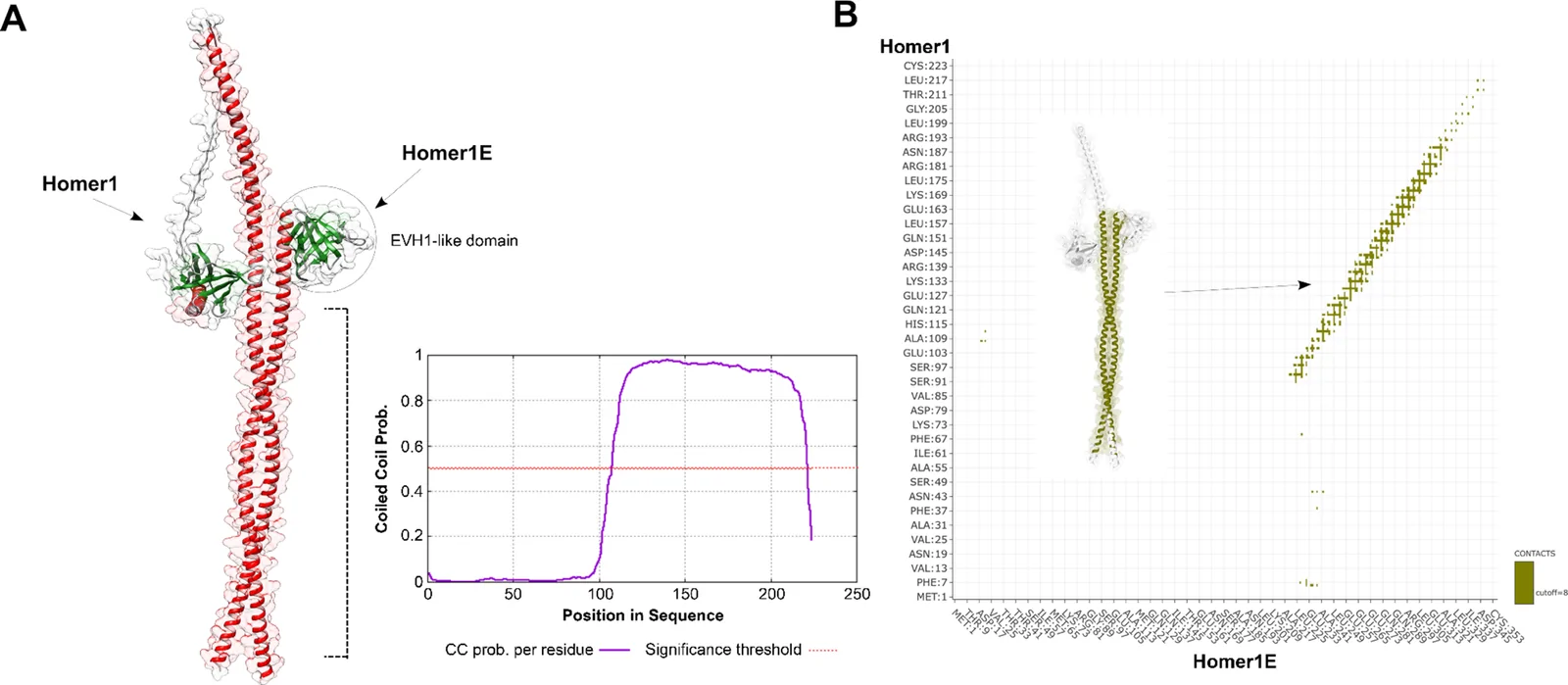
Structural model and interaction analysis of the HOMER1–HOMER1E heterodimer. (**A**) AlphaFold-Multimer model showing the heterodimer formed by HOMER1 and HOMER1E. EVH1-like domains are displayed in green, whereas C-terminal helices form an extended coiled-coil in red (left-hand sided). DeepCoil analysis reveals a strong coiled-coil propensity across the HOMER1E C-terminal region, supporting its ability to participate in helix–helix interactions (right-hand sided). (**B**) Residue-level contact map (8 Å cutoff) between HOMER1 and HOMER1E, highlighting a continuous band of intermolecular contacts characteristic of a stable coiled-coil interface. The inset shows the corresponding region in the structural model

The structural prediction of the uncharacterized intermediate Homer1Er1E (UniProt: Q86YM7-2), generated using AlphaFold (Jumper et al. 2021), revealed a compact EVH1-like N-terminal domain followed by an extended α-helical segment (Fig. 11A). The confidence of the predicted model was assessed using the pLDDT and PAE, which are reported in Fig. 1S. HOMER1 isoforms contain a well-established C-terminal coiled-coil that is responsible for higher-order multimerization, whereas the corresponding region in HOMER1E is truncated yet maintains striking helical continuity. Based on this observation, we hypothesized that the long C-terminal helix may have a latent propensity to engage in coiled-coil interactions despite its limited sequence length.

Thus, DeepCoil (Gabler et al. 2020) was used to predict coiled-coil motifs, which identified significant coiled-coil propensities across the C-terminal helix of HOMER1E (Fig. 11B). The predicted coil region showed significant overlap with the corresponding motif in HOMER1 (UniProt: Q86YM7), indicating that HOMER1E may form intermolecular interactions via this conserved structural element. Consistent with the experimentally determined architecture of the HOMER coiled-coil domain (Hayashi et al. 2009), AlphaFold Server generated a structurally plausible antiparallel HOMER1–HOMER1E heterodimer (Abramson et al. 2024), i.e., an antiparallel coiled-coil assembly consistent with typical HOMER family dimerization patterns. The interface comprises a series of hydrophobic and electrostatic contacts that resemble a heptad-like pattern, supporting the feasibility of a mixed coiled-coil arrangement and suggesting that the shorter HOMER1E isoform remains structurally competent for dimer formation.

## Discussion

The present paper describes a novel HOMER1 transcript expressed in human skeletal muscles, both SOL and VL, i.e., prevalently slow-twitch and fast-twitch muscles, respectively. Interrogation of the human HOMER1 gene identified three validated protein isoforms expressed in skeletal muscle: the long canonical isoform, here designated HOMER1; the short isoform HOMER1H (Transcript Variant 3), which lacks coding exons 6–8 in frameshift and therefore possesses a shorter, distinct C-terminus lacking the intact CC multimerization domain — the human counterpart of the murine short isoform HOMER1a; and a previously uncharacterized intermediate isoform, HOMER1E (Transcript Variant 2), which lacks the in-frame coding exons 4–6 and retains both native termini but lacks the internal autoinhibitory P-motif. For clarity, this nomenclature is used consistently throughout, with HOMER1H referred to as the human short (dominant-negative-type) isoform and HOMER1E as the intermediate isoform.

HOMER proteins act as key regulators of the multimeric complexes involved in several pathways of signal transduction. CC-containing HOMER isoforms couple membrane receptors to intracellular Ca²⁺-release channels, whereas short isoforms lacking the CC domain — such as murine HOMER1a and, by analogy, human HOMER1H — disrupt hetero-multimeric complexes and act as natural dominant negatives. In skeletal muscle, the long isoform HOMER1 associates with RyR1 and modulates its gating and Ca²⁺-release properties (Pettersen et al. 2004, Szumlinski et al. 2006, Tu et al. 1998); against this background, isoforms that modify the composition or architecture of the HOMER1 scaffold would be expected to influence Ca²⁺ release regulation. The present findings are discussed within this functional context.

The three isoforms identified here are predicted to occupy distinct functional positions relative to the HOMER1 scaffold, according to their domain composition. The long HOMER1 isoform, endowed with an intact CC/leucine-zipper domain, is the scaffold-forming isoform: it self-assembles into higher-order multimers and cross-links EVH1-bound target proteins, consistent with its established role at the Ca²⁺-release machinery in skeletal muscle. HOMER1H, which retains the N-terminal EVH1 target-binding domain but lacks the intact CC domain, cannot multimerize; like the murine short isoform HOMER1a, it is therefore predicted to act as a dominant-negative regulator that competes for EVH1 targets and disrupts long-HOMER1 clusters. HOMER1E occupies an intermediate position: it retains a shortened but structurally continuous CC segment (Fig. 11) that supports heterodimerization with HOMER1, and is therefore predicted to act not as a simple disruptor but as a scaffold modulator.

Due to the lack of structural information for the intermediate HOMER1E isoform, structural bioinformatics approaches were employed to generate the model shown in Fig. 11. The predicted structure is consistent with the experimentally observed interaction between HOMER1 and HOMER1E and indicates that the truncated isoform retains a C-terminal coiled-coil compatible with heterodimer formation. Crystallographic studies of the canonical HOMER coiled-coil domain have shown that antiparallel dimers further assemble into tetrameric scaffolds (Hayashi et al. 2009). Our structural analysis focused on evaluating whether the truncated HOMER1E isoform retains compatibility with the canonical coiled-coil dimerization interface. Whether HOMER1E can also participate in mixed tetrameric assemblies or self-associate through its retained coiled-coil region remains an open question requiring dedicated structural and biochemical investigation. If incorporated into higher-order HOMER assemblies, HOMER1E could potentially modulate scaffold architecture by altering the composition of coiled-coil-mediated oligomers. However, this possibility remains speculative and was not directly addressed in the present study.

The physical interaction between HOMER1E and HOMER1, supported by both co-affinity purification data (Figs. 8 and 9) and structural modelling (Fig. 11), provides the mechanistic basis for understanding both the functional role of HOMER1E and its pronounced instability under basal conditions. As judged by ddPCR, HOMER1E is a very minor component of HOMER1 transcripts under basal conditions, and its translation product, at least in HEK293 cells, is highly unstable owing to active autophagic degradation. The pronounced instability of HOMER1E may be a direct structural consequence of the loss of exons 4–6. In full-length HOMER1, the proline-rich autoinhibitory P-motif encoded by this region binds the EVH1 domain at a site partly overlapping that used for Pro-Pro-x-x-Phe ligand binding (Hwang et al. 2003, Shiraishi et al. 2004), thereby engaging and conformationally constraining the EVH1 domain. In HOMER1E this motif is absent, so the EVH1 domain is left without its intramolecular binding partner; we speculate that the resulting unconstrained, “open” conformation, together with the non-native junction created by the internal deletion, renders the isolated HOMER1E protein conformationally unstable and exposes it to recognition by the cellular protein quality-control machinery, leading to its rapid autophagic clearance. The present data do not establish a direct causal relationship between loss of the autoinhibitory P-motif and HOMER1E degradation, and this mechanism remains to be experimentally tested. Moreover, the missing central region may contribute additional intrinsic fold stability, independently of the autoinhibitory P-motif.

Consistent with this interpretation, co-expression with full-length HOMER1 markedly protects HOMER1E from degradation (Figs. 8 and 9). This stabilizing effect is readily explained by the heterodimerization data: by providing a CC-mediated interaction partner, HOMER1 can incorporate HOMER1E into a higher-order scaffold, presumably restoring a more stable conformational environment for the otherwise unconstrained HOMER1E protein. The lack of specific binding sites on HOMER1E may additionally eliminate specific binding partners present in full-length HOMER1, further altering the intrinsic organization of the scaffold. These observations together suggest that the stability and function of HOMER1E are critically dependent on its interaction with canonical HOMER1.

Given its pronounced instability under basal conditions and its stabilization by HOMER1, HOMER1E may exert its modulatory function transiently and/or during specific physiological conditions such as development (Bortoloso et al. 2006). The minuscule amount of mRNA under basal conditions and the rapid turnover of the HOMER1E protein, as shown by heterologous expression experiments, suggest that HOMER1E may affect multimeric HOMER1 scaffolding when expressed in a phasic manner. Within the three-isoform framework outlined above, HOMER1E could conceivably fine-tune multimeric architecture, representing a potential mechanism for adjusting HOMER1 organization under specific physiological conditions — distinct from the disruptive, dominant-negative action predicted for HOMER1H.

Direct immunolocalization of HOMER1H was outside the scope of the present study, which focused on the newly identified HOMER1E; its intracellular distribution in human muscle therefore remains to be determined experimentally. However, the subcellular localization of the equivalent short isoform, HOMER1a, has been characterized in transgenic rat SOL fibers by confocal microscopy: HOMER1a labelled both the I band and the A band, with distinct reinforcement of the H line, and — like HOMER1 — did not colocalize with the SR markers calsequestrin and RyR1 (Volpe et al. 2004). By analogy, HOMER1H would be expected to display a similar sarcomeric, non-triadic distribution in skeletal muscle. Functionally, because HOMER1H lacks the dimerization motif, it is predicted to antagonize the multimeric HOMER1 scaffold — reducing HOMER1-dependent regulation of RyR1/IP₃R signaling — in the same manner attributed to HOMER1a, in contrast to the modulatory role proposed here for HOMER1E.

Overall, the present data support a model in which HOMER1E may act as a context-dependent modulator of HOMER1 scaffold organization, whose activity appears to be tightly regulated both transcriptionally and post-translationally. It remains to be determined which stimuli may upregulate HOMER1E transcription and how HOMER1E regulates Ca²⁺ release from the SR in skeletal muscle.

## Supplementary Information

Below is the link to the electronic supplementary material.

Supplementary Material 1

## Data Availability

No datasets were generated or analysed during the current study.

## Abbreviations

Baf: Bafilomycin A1
CBL: Cerebellum
CBR: Brain
CC: Coiled-Coil
CRH1: Conserved region of HOMER1
EC coupling: Excitation-Contraction coupling
EDL: *Extensor Digitorum Longus*
Ena/VASP: Enabled/vasodilator-stimulated phosphoprotein
ddPCR: Droplet digital PCR
DHPR: Dihydropyridine receptor
FKBP12: FK506 binding protein 12
IEG: Immediate early gene
IP3R: Inositol-trisphosphate receptor
mGluR1: Metabotropic glutamatergic receptor 1
PAE: Predicted Aligned Error
PBS: Phopshate buffer saline
PCR: Polymerase chain reaction
pLDDT: Predicted Local Distance Difference Test
Po: Channel open probability
RYR1: Ryanodine Receptor 1
SR: Sarcoplamic Reticulum
SOL: *Soleus*
TRPC1: Transient Receptor Potential Cation Channel 1
VL: Vastus lateralis
WB: Western Blot
