# Supplementary material for: Cloning and characterization of intermediate Homer1E of human skeletal muscle
Source: J Muscle Res Cell Motil. 2026 Aug 27;47(3):20. doi: 10.1007/s10974-026-09738-x (PMC13522067; doi:10.1007/s10974-026-09738-x)
Supplement: Supplementary file 1 [file 10974_2026_9738_MOESM1_ESM.docx]

**Supplementary Material**

**Figure 1S**

Confidence assessment of the AlphaFold Server prediction for the Homer1–Homer1E heterodimer.

(**A**) Structural model of the HOMER1–HOMER1E heterodimer coloured according to the AlphaFold pLDDT confidence score. Blue and cyan indicate high confidence, whereas yellow and orange indicate regions of lower confidence, largely confined to the terminal segments. (**B**) PAE matrix of the same model. Lower PAE values (dark green) indicate higher confidence in the relative position of residues, whereas higher values (lighter green) reflect increased uncertainty. Overall, the confidence metrics support a well-defined local fold for the EVH1 domains and the coiled-coil region, whereas greater conformational flexibility is expected at the terminal regions.

**Figure 2S**

(**A**) Human *HOMER1* RT-PCR products amplification using adult human cerebellum from autopsy (8 ng) and SOL skeletal muscle (16 ng) cDNA. Sizes of the expected bands are reported on the bottom. First lane reports the 1kb ladder. (**B**) Mouse *HOMER1* RT-PCR products amplification using brain, cerebellum and EDL and SOL skeletal muscle (8 ng) cDNA. For *Homer1M*, PCR was repeated for 45 cycles under the same experimental conditions.

**Figure 3S**

Full-length sequencing of HOMER1E (red) and its comparison to HOMER1. Primers used for PCR and sequencing are indicated in Yellow (FW primers) and green (RV primers).

**FIGURE 4S**

Confocal Immunofluorescence of transfected myogenic (AB678) cells. (**A**) AB678 cells transfected with

cDNAs encoding HOMER were labelled with anti-His-tag antibody and visualized with Alexa Fluor 488 secondary antibody. Scale bars, 10 μm. (**B**) AB678 cells transfected with cDNAs encoding HOMER1E labelled with anti-HA-tag antibody and visualized with an Alexa Fluor 488 conjugated secondary antibody. Scale bars, 10 μm. (**C**) AB678 cells co-transfected with equimolar amounts of cDNAs encoding either HOMER1 or HOMER1E. HOMER1 was labelled with anti-HIS-tag antibody and visualized with Alexa Fluor 488 conjugated secondary antibody; HOMER1E was labelled with anti-HA-tag antibody and visualized with Alexa Fluor 568 conjugated secondary antibody. Nuclei were counterstained with DAPI. Scale bars, 10 μm.

**Table 1S**

Oligonucleotide primers used for PCR, ddPCR experiments and sequencing on human samples

**Table 2S**

Oligonucleotide primers used for PCR on mouse samples
